# Correlations between the sagittal plane parameters of the spine and pelvis and lumbar disc degeneration

**DOI:** 10.1186/s13018-018-0838-6

**Published:** 2018-06-04

**Authors:** Xu Wei, Li Gengwu, Chen Chao, Li Yifan, Sang Shang, Hu Ruixi, Ji Yunhan, Zhu Xiaodong, Li Zhikun

**Affiliations:** 1 Department of Orthopedics, Tongren Hospital, Shanghai Jiao Tong University School of Medicine, 1111 XianXia Road, Shanghai, 200336 People’s Republic of China; 2 Department of Orthopedics, Panzhihua Central Hospital, 34 YiKang Road, Panzhihua, 617017 Sichuan Province People’s Republic of China

**Keywords:** Intervertebral disc degeneration, Disc herniation, Spinal-pelvic parameters, Sagittal plane balance

## Abstract

**Background:**

Studies have shown that lumbar disc herniation, degenerative lumbar instability, and other degenerative lumbar spinal diseases are often secondary to disc degeneration. By studying the intervertebral disc, researchers have clarified the pathological changes involved in intervertebral disc degeneration but have ignored the roles of biomechanical factors in the development of disc degeneration. This study aims to investigate the relationships among the location, scope, and extent of lumbar disc degeneration and sagittal spinal-pelvic parameters.

**Methods:**

A retrospective analysis was performed on the clinical data of 284 patients with lumbar degenerative disc diseases (lumbar disc herniation and degenerative lumbar instability) from January 2013 to December 2016. Statistics were calculated for the following: (1.) patients’ general information: name, sex, age, height, and weight. (2.) Measurements of sagittal parameters from total spinal radiographs: thoracic kyphosis (TK), Lumbar lordosis (LL), sacral slope (SS), pelvic tilt (PT), pelvic incidence (PI), sagittal vertical axis (SVA), T1 tilt angle (TA), and T1 pelvic angle (TPA). (3.) Location, scope, extent, and overall degree of lumbar disc degeneration. Parameters were analyzed in groups by sex, PI, and SVA, and a correlation analysis was performed for the location, scope, extent, and overall degree of lumbar intervertebral disc degeneration with 8 spinal-pelvic sagittal parameters.

**Results:**

The mean ages of the male and female patient groups were 59.00 and 53.28 years old, respectively (*P* < 0.05). The PT, location, scope, and overall degree of degradation were significantly different between the sexes (*P* < 0.05). Linear correlation analysis results showed that the overall degree and extent of degradation (*r* = 0.788, *P* < 0.01), LL and SS (*r* = 0.737, *P* < 0.01), PI and PT (*r* = 0.607, *P* < 0.01), and TPA and PT (*r* = 0.899, *P* < 0.01) were strongly correlated. The location values were 4.08 ± 0.72 in patients with PI≤50° and 3.62 ± 0.94 in patients with PI> 50° (*P* = 0.018). Different SVASVA groups differed in their overall degree of degeneration (*P* = 0.002).

**Conclusions:**

The location of lumbar intervertebral disc degeneration is affected by spinal-pelvic sagittal morphology. Populations with small PI values tend to exhibit degeneration at the L4/5 and L5/S1 discs, and populations with large PI values tend to exhibit degeneration at the L3/4 and L4/5 discs. The SVA value and the overall degree of lumbar disc degeneration are positively correlated.

## Background

The lumbar spine is the hub of human torso activity. Increasing age, excessive activity, and overloading may cause accelerated aging of the lumbar vertebrae, and external forces may cause secondary pathological changes leading to the rupture of the intervertebral disc annulus fibrous, prolapsed intervertebral disc nucleus pulposus, and lower back pain and neurological dysfunction [1]. Lumbar degenerative diseases include the degeneration of the intervertebral disc, cartilage end plate, vertebral body, and ligaments, of which the degeneration of intervertebral discs is the focus of our attention [2].

Studies have shown that lumbar disc herniation, degenerative lumbar instability, and other degenerative lumbar spinal diseases, such as hyperplasia of articular processes, wedging of vertebral bodies, and hyperosteogeny, are often secondary to disc degeneration. Intervertebral disc degeneration is related to many factors, including spine biomechanics, biology, injury, inflammation, and nutrition [3–6]. By studying the intervertebral disc, researchers have clarified the pathological changes involved in intervertebral disc degeneration but have ignored the roles of biomechanical factors during its development [7]. When a person is maintaining a standing position, various parts of the body must be in coordination, forming different spine sagittal patterns and resulting in different biomechanical characteristics [8, 9]. Previous studies have indicated that there are changes in the spinal-pelvic sagittal force lines in patients with spinal deformities and lumbar degenerative diseases to varying degrees [10]. To date, research on whether lumbar intervertebral disc degeneration is related to differences in spinal-pelvic sagittal morphology has been sparse. In this study, correlations of the location, scope, and extent of lumbar intervertebral disc degeneration with sagittal spinal and pelvic parameters were investigated.

## Methods

### General information

This retrospective study was approved by the Ethics Committee of Shanghai Tongren Hospital. The study subjects were patients with lumbar disc degenerative disease admitted to Shanghai Tongren Hospital from January 2013 to December 2016. Inclusion criteria were the following: (1) age > 18 years old, with a clear diagnosis of lumbar disc degenerative disease [11] (lumbar disc herniation and degenerative lumbar instability, diagnostic criteria referring to NASS evidence-based clinical guidelines, and imaging findings matching clinical manifestations [11]) and (2) patients with full spinal anteroposterior and lateral radiographs and preoperative lumbar MRIs. Exclusion criteria were the following: (1) patients with spinal tumors, trauma, inflammation, isthmus, or deformities; and (2) patients with neuromuscular disorders or lower limb diseases affecting their ability to stand. Statistical analysis was performed to analyze patient age, height, and weight.

### Image measurement

Using a picture-archiving and communication system (PACS), spinal-pelvic sagittal parameters over the full spine in anteroposterior and lateral radiographs were measured using the maximum Cobb angle method [12–14]. Six intervertebral discs between T12 and S1 were evaluated on a T2-weighted MRI image of the lumbar spine. The specific methods used are described in the following sections.

#### Spinal-pelvic parameters

(1) Thoracic kyphosis (TK) is the vertebral body with the largest upper thoracic tilt to the junction of the thoracic kyphosis and lumbar lordosis. (2) Lumbar lordosis (LL) is the junction of the thoracic kyphosis and lumbar lordosis to the S1 end plate. (3) Pelvic incidence (PI) is the angle between the line perpendicular to the S1 end plate from the midpoint of the end plate and the connecting line from the midpoint of the S1 end plate to the center of the femoral head. (4) Pelvic tilt (PT) is the angle between the connecting line from the midpoint of the S1 end plate to the center of the femoral head and the vertical line. (5) Sacral slope (SS) is the angle between the tangent and the horizontal line of the S1 end plate. (6) Sagittal vertical axis (SVA) is the horizontal distance between the vertical line of the C7 vertebral center and the posterior upper angle of the S1 end plate. (7) T1 tilt angle (TA) is the angle between the T1 vertebral end plate and the horizontal line. (8) T1 pelvic angle (TPA) is the angle between the connecting line from the T1 midpoint to the femoral head centerline and the connecting line from the sacral end plate midpoint to the center of the femoral head.

**Fig. 1:**
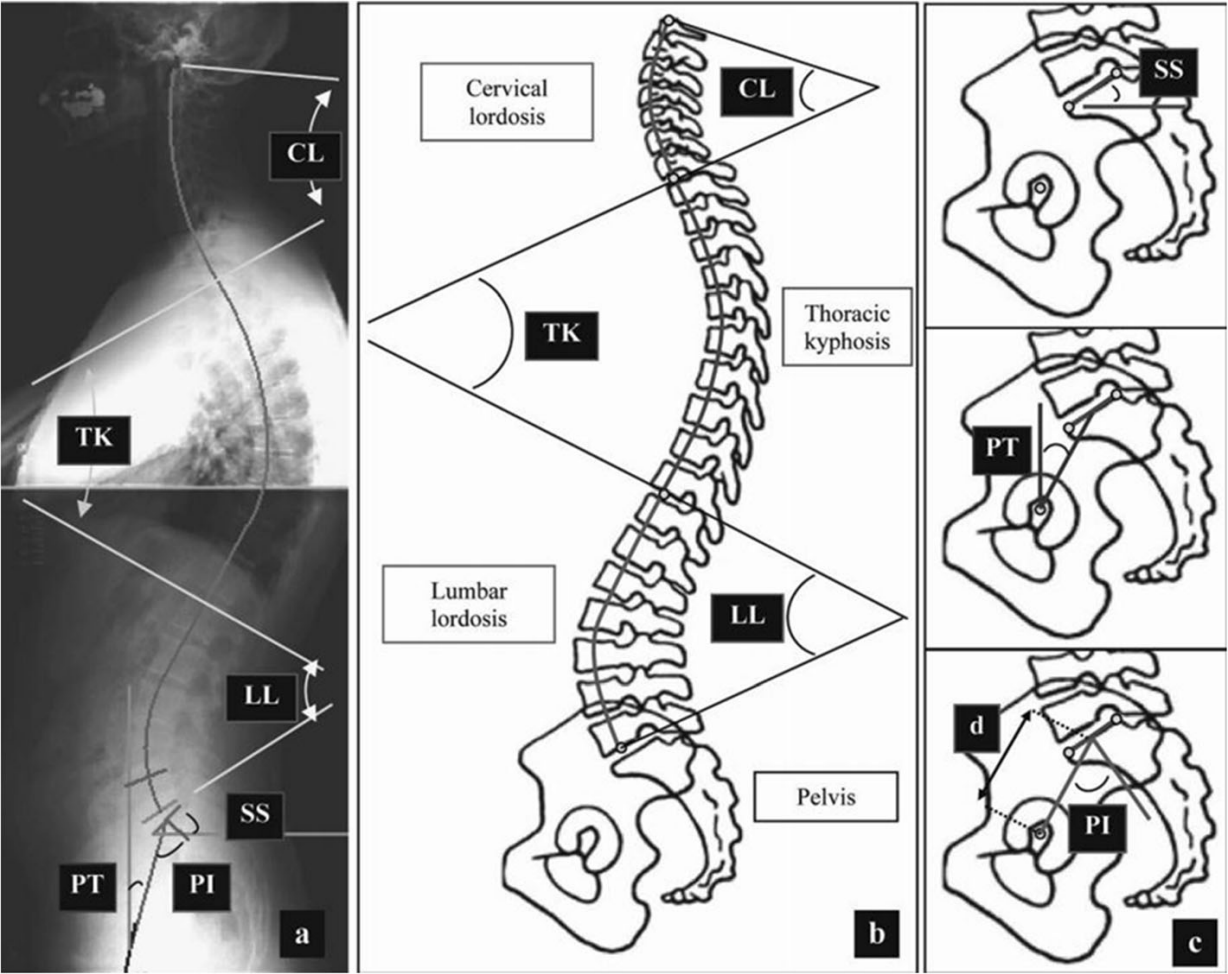
Detailed definitions of spinal parameters

Detailed definitions are provided in Fig. 1 [15].

#### Grading of disc degeneration

The Pfirrmann grading system [16] was used with the following definitions. Grade 1, the nucleus pulposus is homogeneous and translucent, with a clear boundary from the annulus fibrosus, and the height of the intervertebral disc is normal. Grade 2, the nucleus pulposus is not fully homogeneous, with a clear boundary from the annulus fibrosus, and the height of the intervertebral disc is normal. Grade 3, the nucleus pulposus is moderately darkened, with an unclear boundary from the annulus fibrosus, and the height of the intervertebral disc is normal or slightly lowered. Grade 4, a black disc is observed, there is no boundary between the nucleus pulposus and the annulus fibrous, and the height of the intervertebral disc is less than normal. Grade 5, a black disc is observed, there is no boundary between the nucleus pulposus and the annulus fibrous, and the intervertebral space has collapsed. The degeneration grade can directly reflect the severity of intervertebral disc degeneration: grade 1 was recorded as 1, grade 2 as 2, etc. Grades 1–5 correspond to A–E in the following chart.

**Fig. 2:**
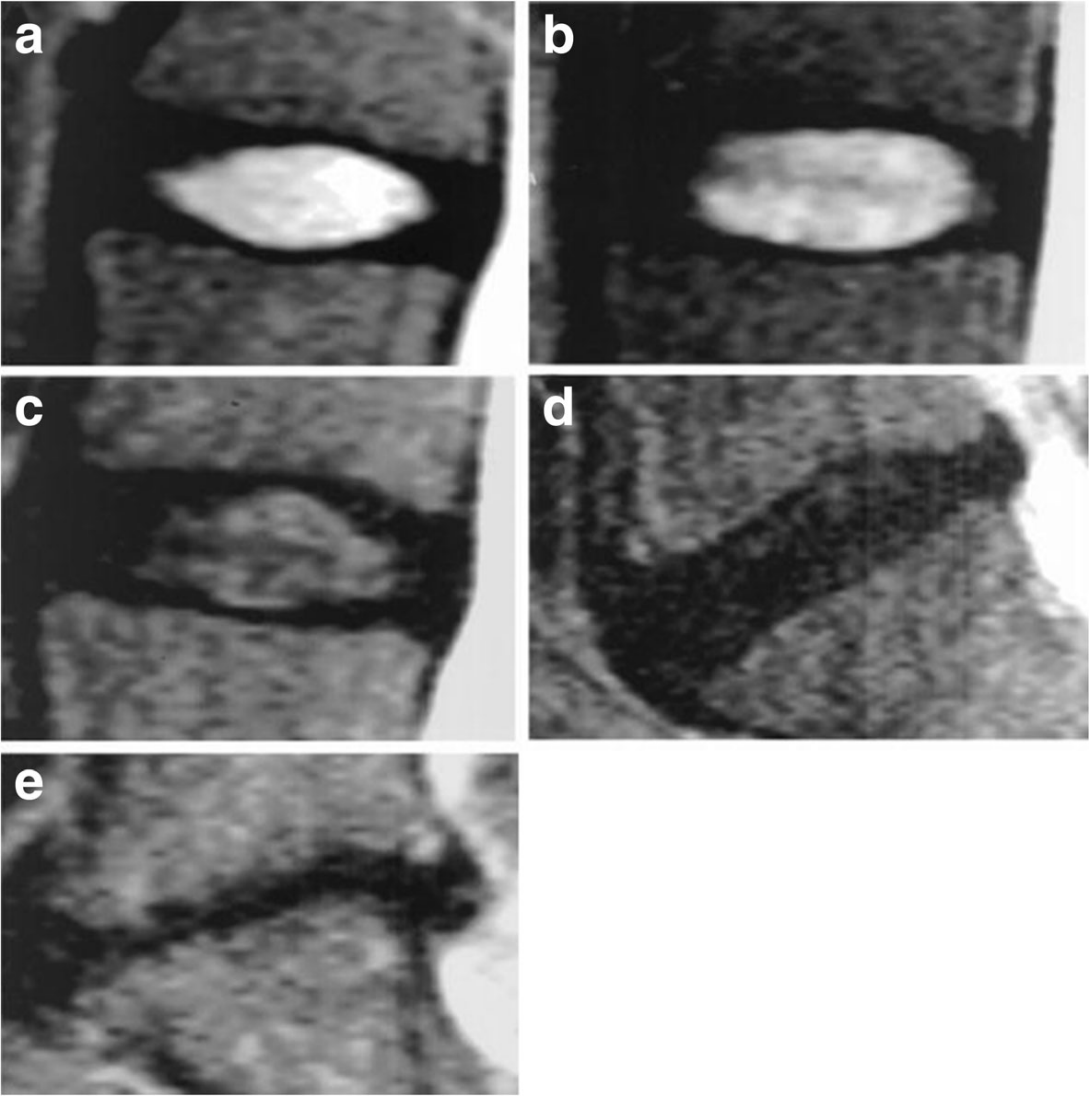
Detailed definitions of grading classification. The Pfirrmann grading system: Grade **a**-**e**

Grading is shown in Fig. 2 [16].

### Location quantization, scope selection, and the extent of the degenerative process

(1) Location quantification: the discs from T12 to S1 were recorded as 0, 1, 2, 3, 4, and 5, respectively. The degeneration at intervertebral disc T12/L1 was recorded as 0; the degeneration at intervertebral disc L1/2 was recorded as 1; the degeneration at intervertebral disc L2/3 was recorded as 2; the degeneration at intervertebral disc L3/4 was recorded as 3; the degeneration at intervertebral disc L4/5 was recorded as 4; and the degeneration at intervertebral disc L5/S1 was recorded as 5. The numerical value reflects the location of the intervertebral disc and matches the number of the intervertebral disc. (2) Scope selection (the number of intervertebral discs): grades 4 and 5 intervertebral disc segments according to Pfirrmann grading were selected. If the most serious segment was grade 3, then the corresponding grade 3 segment was selected. (3) Extent of the degenerative process: the extent of degeneration of the intervertebral disc within the scope of overall degeneration were summed and then divided by the scope of overall degeneration.

Example:

Calculation of degenerative location: if the degeneration at L2, L3, and L4 was greater than grade 3, then the degenerative location was calculated as (2 + 3 + 4)/3 = 3.

Calculation of degenerative scope: If the highest level of lumbar intervertebral disc degeneration was 3, then the number of the 3-stage degenerative disc was recorded. For example, L1/2, L2/3, L3/4, and L4/5 are level 2, and L5/S1 is level 3. The degenerative scope was calculated as 1. If the highest level of lumbar intervertebral disc degeneration was 4 or 5, then the number of degenerative discs in the 4 or 5 levels was recorded. For example, L1/2, L2/3, and L3/4 are 3, L4/5 is 4, and L5/S1 is 5. The degenerative scope was calculated as 2. Calculation of the extent of degeneration: if the degeneration at L4/5 was grade 3, and the degeneration at L5/S1 was at grade 5, then the average extent of degeneration was (3 + 5)/2 = 4. The overall degree of degradation is the sum of the extent of degeneration of each segment.

### Statistical analysis

Data collection and statistical analyses were performed using the statistical software SPSS 21.0 (Chicago, IL, USA). Descriptive statistical analysis was performed for each parameter, and the differences in each parameter between sexes were compared with a bilateral independent samples *t* test. Differences with *P* < 0.05 were considered statistically significant. Pearson correlation analysis (bilateral) was performed for the location, scope, and extent of intervertebral disc degeneration with age, BMI, and sagittal spinal-pelvic parameters. Correlations with a *P* value of *P* < 0.05 were considered significant. Based on the different groupings of TK, LL, SS, PT, PI, SVA, TA, and TPA, the location, scope, extent, and overall degree of intervertebral disc degeneration were analyzed using bilateral independent t tests. Differences with *P* < 0.05 were considered statistically significant.

## Results

**Fig. 3:**
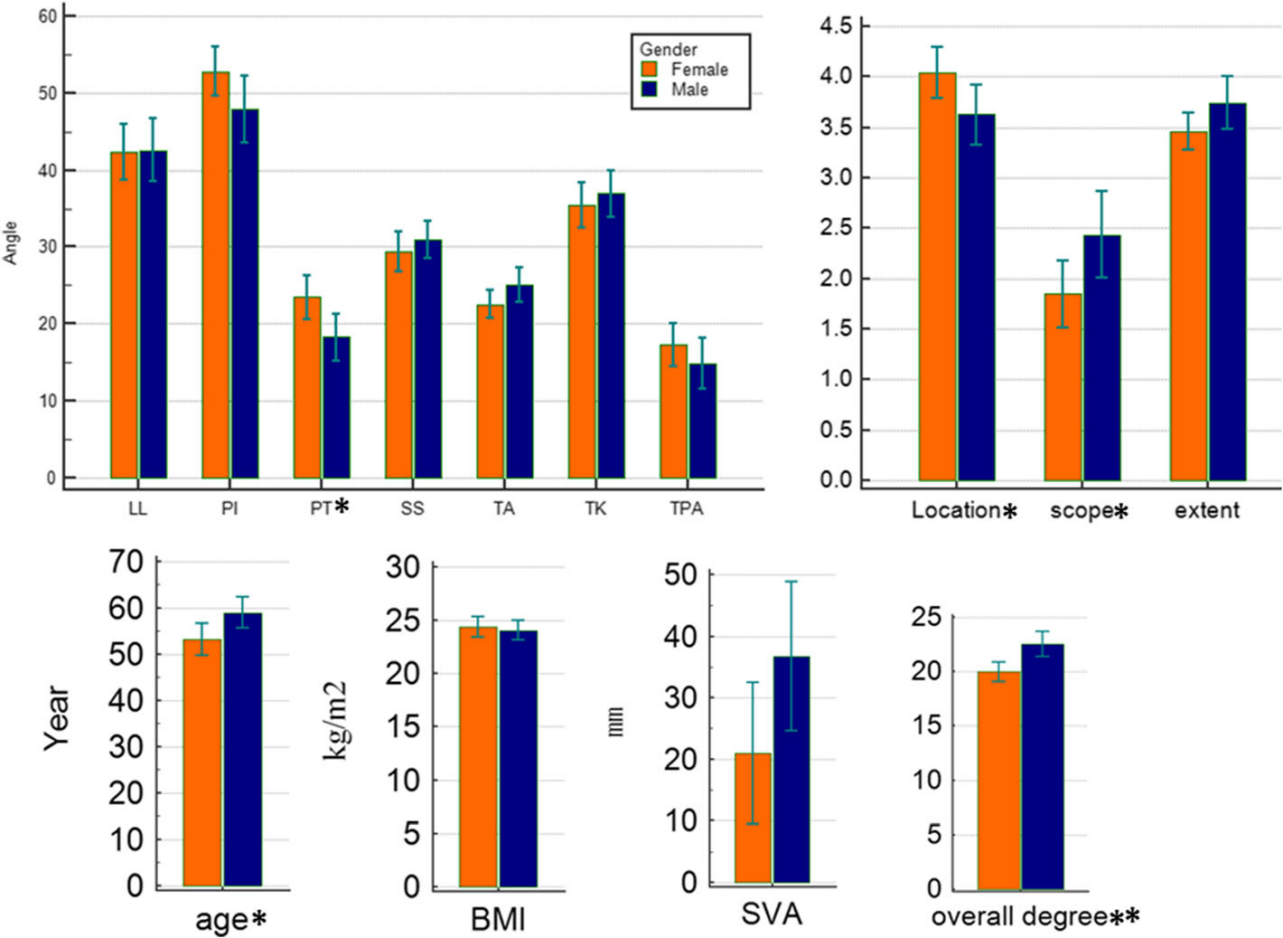
Bar charts between males and females for each parameter are shown. *P < 0.05, significant correlation; **P < 0.01, significant correlation

The 284 patients from Shanghai Tongren Hospital in this study included 141 males and 143 females, with ages ranging from 19 to 78 years and a mean age of 56.14 years. The mean ages of the male and female patients were 59.00 ± 10.347 and 53.28 ± 10.521 years (*P* < 0.05), respectively, as shown in Table 1 and Fig. 3.

**Table 1:** The mean values, standard deviation and differences between males and females on each parameter

	*n* = 284	Male (*n* = 141)	Female (*n* = 143)	*P*
Age (year)	56.14 ± 10.76	59.00 ± 10.347	53.28 ± 10.521	0.018*
BMI (kg/m2)	24.24 ± 2.93	24.076 ± 2.90	24.406 ± 2.98	0.623
TK (°)	36.19 ± 9.25	36.92 ± 9.551	35.46 ± 9.011	0.489
LL (°)	42.47 ± 11.79	42.59 ± 12.684	42.36 ± 10.996	0.932
PI (°)	50.35 ± 12.00	47.885 ± 13.314	52.821 ± 10.110	0.069
SS (°)	30.19 ± 7.64	31.00 ± 7.483	29.38 ± 7.795	0.353
PT (°)	20.86 ± 9.29	18.28 ± 9.313	23.44 ± 8.629	0.013*
SVA (mm)	28.90 ± 37.09	36.77 ± 37.372	21.03 ± 35.549	0.060
TA (°)	23.79 ± 6.47	25.03 ± 7.054	22.56 ± 5.651	0.093
TPA (°)	16.06 ± 9.36	14.85 ± 10.114	17.28 ± 8.494	0.253
Location	3.83 ± 0.87	3.628 ± 0.916	4.038 ± 0.790	0.037*
Scope	2.14 ± 1.21	2.44 ± 1.334	1.85 ± 1.014	0.031 *
Extent	3.60 ± 0.69	3.74 ± 0.785	3.46 ± 0.555	0.071
Overall degree	21.27 ± 3.42	22.54 ± 3.582	20.00 ± 2.753	0.001**

Note: *t* test (bilateral); *P < 0.05; **P < 0.01; the data in the Table are presented as the mean ± SD

The results of the correlation analysis are shown in Table 2, in which the positive correlations between the overall degree and the extent of degeneration (*r* = 0.788, *P* < 0.01), SS and LL (*r* = 0.737, *P* < 0.01), PT and PI (*r* = 0.607, *P* < 0.01), and TPA and PT (*r* = 0.899, *P* < 0.01) were strong.

**Table 2:** The correlations between the sagittal parameters and the location of the lumbar disc degeneration, correlation coefficient

	Age	BMI	Location	Scope	Extent	Overall degree	TA	TK	LL	PI	SS	PT	SVA	TPA
Age	1	− 0.88	− 0.343**	0.339**	0.415**	0.486**	0.182	0.352**	0.161	0.033	− 0.001	0.052	0.190	0.120
BMI		1	− 0.134	0.039	0.198	0.077	− 0.021	− 0.038	− 0.037	0.064	− 0.050	0.138	0.023	0.141
Location			1	− 0.541**	− 0.445**	− 0.471**	− 0.264*	− 0.100	0.012	− 0.150	− 0.096	− 0.264*	− 0.154	− 0.330**
Scope				1	0.316**	0.494**	0.121	0.105	0.024	0.119	0.018	0.193	0.285*	0.270*
Extent					1	0.788**	0.211	0.132	− 0.037	− 0.075	− 0.003	0.060	0.165	0.155
Overall degree						1	0.208	0.190	−0.037	−0.126	−0.057	0.047	0.375**	0.230*
TA							1	0.583**	− 0.135	−0.203	−0.037	0.014	0.324**	0.182
TK								1	0.535**	− 0.017	0.111	− 0.131	−0.046	− 0.099
LL									1	0.428**	0.737**	−0.256*	−0.412**	−0.379**
PI										1	0.416**	0.607**	0.078	0.416**
SS											1	− 0.208	− 0.338**	− 0.259*
PT												1	0.283*	0.899**
SVA													1	0.547**
TPA														1

Note: Pearson correlation test (bilateral, *n* = 284); *P < 0.05, significant correlation; **P < 0.01, significant correlation; 0.2–0.4 indicates a weakly positive correlation, 0.4–0.6 indicates a moderate correlation, and 0.6–0.8 indicates a strongly positive correlation

**Fig. 4:**
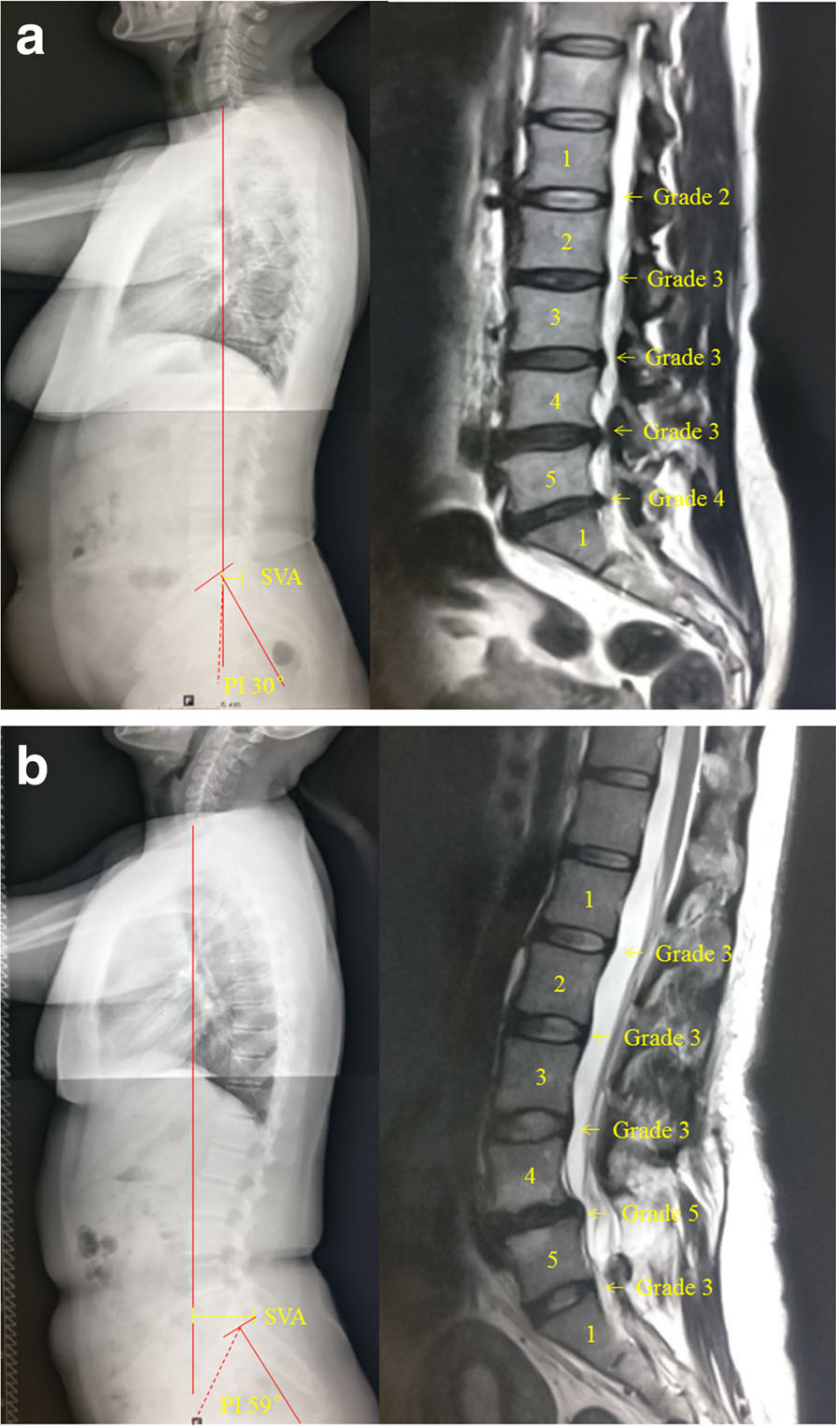
**a** Patients with PI = 35°. **b** Patients with PI = 59°

According to groupings by different PI, the degenerative location in patients with PI≤50° was 4.14 ± 0.64, and the degenerative location in the patients with PI> 50° was 3.57 ± 1.08 (*P* < 0.05). According to groupings by different SVA, the overall degree of degeneration in patients with SVA ≥ 29° was 20.27 ± 2.675, and in patients with SVA > 29°, it was 22.64 ± 3.872 (*P* < 0.01), as shown in Tables 3 and 4, respectively. There were no significant differences between groups in TK, LL, SS, and PT. Representative cases are shown in Fig. 4a, b.

**Table 3:** Relationships among the location, scope, extent of intervertebral disc degeneration, and PI

	PI ≤ 50° (mean ± SD, *n* = 144)	PI > 50° (mean ± SD, *n* = 140)	*P*
Location	4.08 ± 0.72	3.62 ± 0.94	0.018*
Scope	2.00 ± 0.926	2.26 ± 1.415	0.345
Extent	3.50 ± 0.655	3.69 ± 0.715	0.227
Overall degree	20.97 ± 3.211	21.52 ± 3.611	0.481

Note: *t* test (bilateral); *P < 0.05

**Table 4:** The relationship between the location, scope, extent of intervertebral disc degeneration, and SAV

	SAV ≤ 29° (mean ± SD, *n* = 139)	SAV > 29° (mean ± SD, *n* = 145)	*P*
Location	3.978 ± 0.846	3.636 ± 0.886	0.088
Scope	1.93 ± 1.053	2.42 ± 1.370	0.077
Extent	3.53 ± 0.548	3.70 ± 0.847	0.304
Overall degree	20.27 ± 2.675	22.64 ± 3.872	0.002**

Note: *t* test (bilateral); **P < 0.01;

**Fig. 5:**
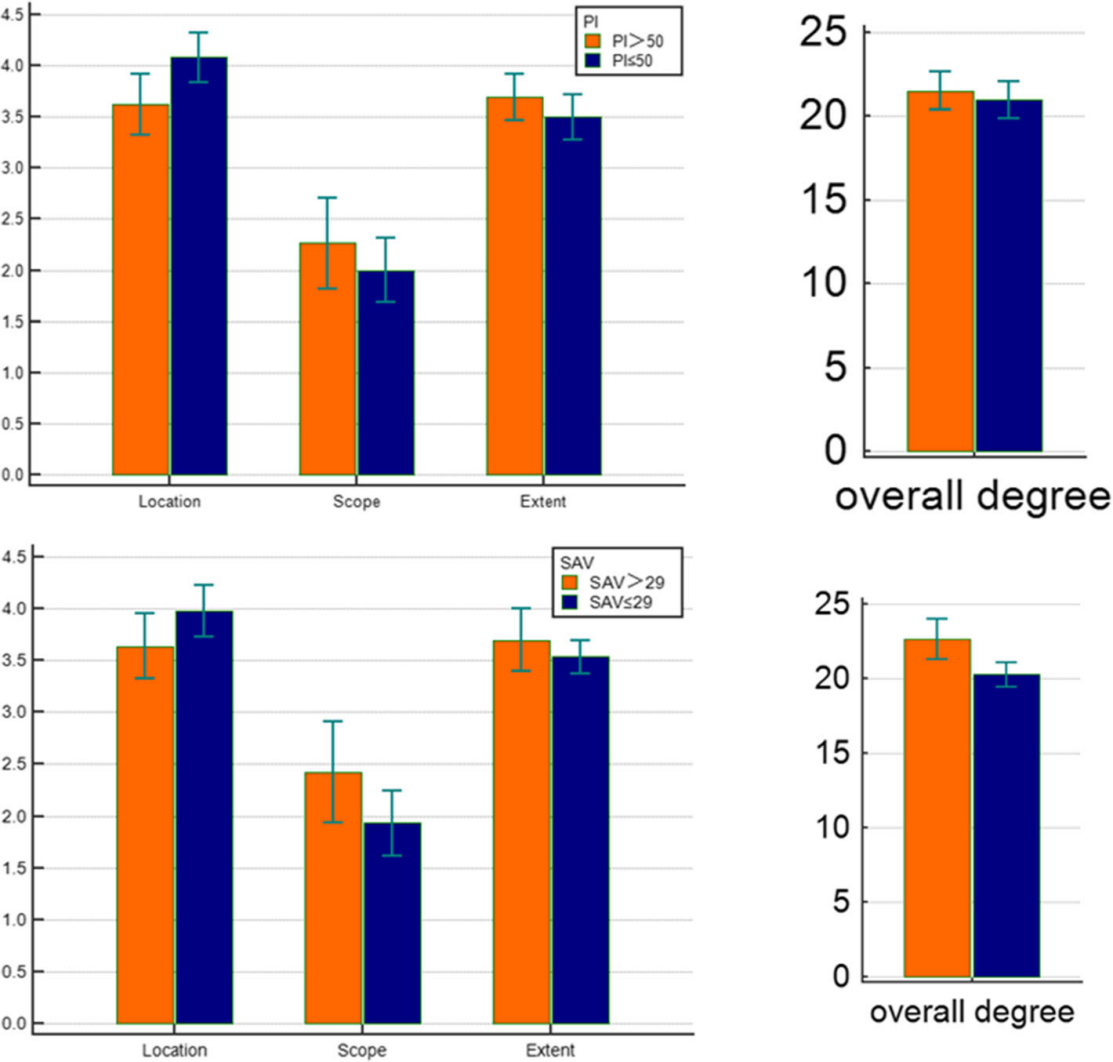
Bar charts between PI> 50 and PI≤50 and between SVA > 29 and SVA ≤ 29 for each parameter are shown. *P < 0.05, significant correlation; **P < 0.01, significant correlation

## Discussion

Lumbar intervertebral disc degeneration is related to various diseases, including lumbar disc herniation, degenerative spondylolisthesis, and degenerative lateral scoliosis. Previous studies have suggested that age is closely related to the degeneration of lumbar intervertebral discs [6, 17]. The results of this study showed that age was closely related to the degenerative location (*r* = − 0.343, *P* < 0.01), the scope of degeneration (*r* = 0.339, *P* < 0.01), and the extent of degeneration (*r* = 0.415, *P* < 0.01). With increased age, the degenerative location was higher, the scope increased, and the extent of degeneration was aggravated. The position and extent of intervertebral disc degeneration were directly proportional to age. Age (*P* = 0.018), PT (*P* = 0.013), location (*P* = 0.037), scope (*P* = 0.031), and degree of degeneration were significantly different between the sexes (*P* = 0.001); compared with females, males had a higher degree of degeneration and a higher scope and age (proportional) but a lower PT and position (inverse).

The impact of stress on lumbar disc degeneration should not be ignored. The lumbar curvature gradually increases from top to bottom. The lower lumbar spine accounts for 2/3 of the entire lumbar lordosis (LL) and receives the most concentrated stress, making it a common segment in which lumbar disc degeneration occurs [18]. Stress plays an important role in the degeneration of the lumbar disc [19]. Biomechanical studies have shown that the degeneration of the lumbar intervertebral disc is related to vertical longitudinal loading and the shearing force on the intervertebral disc [20], and the magnitude of the shearing force varies with spinal movement [7]. Lumbar degeneration may lead to lumbar disc herniation, which primarily occurs in the L4/5 and L5/S1 intervertebral discs [21]. Lumbar degeneration may also lead to lumbar instability, but the segment in which this instability commonly occurs is different from that in which lumbar disc herniation occurs. Aono prospectively investigated 142 females for over 8 years and found an incidence of degenerative spondylolisthesis of 12.7%, including 4 cases of L3 and 14 cases of L4 degenerative spondylolisthesis. Compared with patients with L4 degenerative spondylolisthesis, patients with spondylolisthesis had increased LL, PI, and vertebral tilt and loss of intervertebral height values, and patients with L3 degenerative spondylolisthesis had increased PI, lumbar curvature, and tilting of the L3 vertebrae [22]. Gille et al. reported the analysis of 670 cases of degenerative spondylolisthesis collected from multiple centers in Europe, in which 73% of degenerative spondylolisthesis occurred in the L4/5 intervertebral disc, 18% occurred in the L3/4 intervertebral disc, and 3% occurred in the L2/3 intervertebral disc [23]. Our results showed that the average location of lumbar disc degeneration is 3.83 ± 0.87, which is consistent with the average location of these two typical intervertebral disc diseases. A typical case is shown in Fig. 1.

Different lumbar curvatures may cause changes in biomechanics, and lumbar curvature varies by individual. Roussouly classified lumbar curvature into four types. Type 1: SS is less than 35°, PI is small, the vertex of LL is the lowest of all types, located in the middle of L5, with a minimal lumbar vertebral body, and the thoracolumbar turning point is low and backward, forming a large arcuate thoracic kyphosis. Type 2: SS is less than 35°, the vertex of LL is at the bottom of L4, the lumbar spine is long, the LL contains more of the vertebral body, the thoracolumbar turning point is more ventral than that in type 1, and the overall lumbar curvature and thoracic curvature are relatively small. Type 3: SS is between 35° and 45°, the vertex of LL is at the middle of L4, the lumbar curvature is larger than type 2, and the spinal sagittal plane is in a perfect S shape. Type 4: SS is greater than 45°, the vertex of LL is at L3 or higher, and the lumbar curvature is greatest. Disc herniation is thought to occur easily in types 1 and 2 individuals, spinal stenosis can easily occur in type 4 individuals, and the incidence of lumbar diseases in type 3 individuals is lowest [14]. Chaleat-Valayer proposed that lower back pain is more likely to occur in type 2 individuals, and Roussouly found that type 4 individuals have larger PI values than the other types [24].

The sagittal morphology of a normal spine is closely related to the pelvic parameters [13, 25]. The sagittal morphology (head-spine-pelvis-lower extremities) interacts, and the center of gravity and visual balance are maintained by increasing or decreasing the internal curvature of the spine, the posterior rotation of the pelvis, and the bending of the lower extremities [26, 27]. The results of this study showed that for PI and SS (*r* = 0.416, *P* < 0.01), SS and LL (*r* = 0.428, *P* < 0.01), LL and TK (*r* = 0.535, P < 0.01), SVA and TA (*r* = 0.324, *P* < 0.01), and SVA and the overall degree of degeneration (*r* = 0.375, *P* < 0.01), the correlations with the sagittal plane were consistent with previous findings. With increases in the scope and extent of degeneration, the spine continues to lean forward. Our results showed correlations between age and TK (*r* = 0.352, *P* < 0.01), age and LL (*r* = 0.161, *P* > 0.05); i.e., TK increased with age, while LL did not, which is consistent with the findings of Zhu [25]. However, the result for LL was not consistent with the findings of Xu (age and LL (*r* = − 0.37, *P* = 0.01)) [28]. This relationship requires further clarification.

A literature review found that, among all types of lumbar disc degeneration, patients with herniation exhibited smaller PI values [14] while patients with degenerative lumbar spondylolisthesis exhibited higher PI values [29–31]. The PI size corresponds to differences in the curvature of the lumbar spine. A small PI corresponds to a small lumbar curvature, often forming a sharp corner at L5. A large PI corresponds to a large arc, often including L3, L4, L5, or even more vertebral bodies, and each vertebral inclination is larger. Our results showed that after grouping by PI, the location value was 4.08 ± 0.72 for patients with PI≤50° (the intervertebral disc degeneration of L4/5 and L5/S1), while the location value for patients with PI> 50° was 3.62 ± 0.94 (the intervertebral disc degeneration of L3/4 and L4/5) (*P* < 0.05). The location value was higher in the PI ≤ 50° group than in the PI > 50° group. This phenomenon occurs because in some patients with large PI, the L5/S1 intervertebral disc was more tilted, and the extent of degeneration was significantly lower than that of the L4/5 intervertebral discs, which was considered to be related to the protection of the posterior structure, i.e., the supporting roles of small joints, ligaments, and muscles. SVA was related to the overall degree of degeneration (*P* < 0.01); a larger SVA resulted in a larger PI and a greater degree of degeneration. When the SVA was in the normal range, the PI decreased, and the patients’ degeneration decreased.

The correlations of spinal-pelvic parameters with the location, scope, and extent of degeneration of the lumbar vertebrae not only can predict the degeneration of intervertebral discs in different spinal-pelvic shapes but can also provide healthcare guidance for patients with different PI and SVA values, thus delaying lumbar degeneration, which has never been previously reported. However, this study has some limitations. (1.) Patient lifestyle was not assessed in this study and may affect lumbar degeneration. (2.) The sample size of this study is the minimum mathematically determined sample size; the sample size was not increased due to concerns related to workload. (3.) The study duration was short, and the results must be verified with long-term data. (4.) This is a retrospective study and lacks experimental or mechanical research support. Detailed data are shown in Fig. 5.

## Conclusion

The location of lumbar disc degeneration is affected by the sagittal morphology of the spinal pelvis. Populations with PI≤50° are prone to degeneration at the L4/5 and L5/S1 discs, and populations with PI> 50° are more likely to have degeneration at the L3/4 and L4/5 discs. The position of intervertebral disc degeneration was higher than that of PI> 50 patients, and the SVA value was positively correlated with the overall degree of lumbar disc degeneration.

## Abbreviations

CI: Confidence interval
LL: Lumbar lordosis
MRI: Magnetic resonance imaging
NASS: North American Spine Society
PACS: picture-archiving and communication system
PI: Pelvic incidence
PT: Pelvic tilt
SS: Sacral slope
SVA: Sagittal vertical axis
TK: Thoracic kyphosis

## References

[1] Modic MT, Ross JS (2007). Lumbar degenerative disk disease. Radiology.

[2] Videman T, Battie MC, Ripatti S (2006). Determinants of the progression in lumbar degeneration: a 5-year follow-up study of adult male monozygotic twins. Spine (Phila Pa 1976).

[3] Arun R, Freeman BJ, Scammell BE (2009). 2009 ISSLS prize winner: what influence does sustained mechanical load have on diffusion in the human intervertebral disc?: an in vivo study using serial postcontrast magnetic resonance imaging. Spine (Phila Pa 1976).

[4] Wang YX, Griffith JF (2011). Menopause causes vertebral endplate degeneration and decrease in nutrient diffusion to the intervertebral discs. Med Hypotheses.

[5] Hee HT, Chuah YJ, Tan BH (2011). Vascularization and morphological changes of the endplate after axial compression and distraction of the intervertebral disc. Spine (Phila Pa 1976).

[6] Teraguchi M, Yoshimura N, Hashizume H, et al. Progression, incidence, and risk factors for intervertebral disc degeneration in a longitudinal population-based cohort: the Wakayama spine study. Osteoarthr Cartil. 2017;25:1122–31. 10.1016/j.joca.2017.01.001 28089899

[7] Inoue N, Espinoza Orias AA (2011). Biomechanics of intervertebral disk degeneration. Orthop Clin North Am.

[8] Duval-Beaupere G, Schmidt C, Cosson P (1992). A Barycentremetric study of the sagittal shape of spine and pelvis: the conditions required for an economic standing position. Ann Biomed Eng.

[9] Roussouly P, Pinheiro-Franco JL (2011). Biomechanical analysis of the spino-pelvic organization and adaptation in pathology. Eur Spine J.

[10] Barrey C, Jund J, Noseda O (2007). Sagittal balance of the pelvis-spine complex and lumbar degenerative diseases. A comparative study about 85 cases. Eur Spine J.

[11] Kreiner DS, Hwang SW, Easa JE (2014). An evidence-based clinical guideline for the diagnosis and treatment of lumbar disc herniation with radiculopathy. Spine J.

[12] Mac-Thiong JM, Berthonnaud E, Dimar JR (2004). Sagittal alignment of the spine and pelvis during growth. Spine (Phila Pa 1976).

[13] Vialle R, Levassor N, Rillardon L (2005). Radiographic analysis of the sagittal alignment and balance of the spine in asymptomatic subjects. J Bone Joint Surg Am.

[14] Roussouly P, Gollogly S, Berthonnaud E (2005). Classification of the normal variation in the sagittal alignment of the human lumbar spine and pelvis in the standing position. Spine (Phila Pa 1976).

[15] Berthonnaud E, Labelle H, Roussouly P (2005). A variability study of computerized sagittal spinopelvic radiologic measurements of trunk balance. J Spinal Disord Tech.

[16] Pfirrmann CW, Metzdorf A, Zanetti M (2001). Magnetic resonance classification of lumbar intervertebral disc degeneration. Spine (Phila Pa 1976).

[17] Sivan SS, Wachtel E, Roughley P (2014). Structure, function, aging and turnover of aggrecan in the intervertebral disc. Biochim Biophys Acta.

[18] Korovessis PG, Stamatakis MV, Baikousis AG (1998). Reciprocal angulation of vertebral bodies in the sagittal plane in an asymptomatic Greek population. Spine (Phila Pa 1976).

[19] Hirsch C (1955). The reaction of intervertebral discs to compression forces. J Bone Joint Surg Am.

[20] Stokes IA, Iatridis JC (2004). Mechanical conditions that accelerate intervertebral disc degeneration: overload versus immobilization. Spine (Phila Pa 1976).

[21] Deyo RA, Mirza SK (2016). CLINICAL PRACTICE. Herniated Lumbar Intervertebral Disk. N Engl J Med.

[22] Aono K, Kobayashi T, Jimbo S (2010). Radiographic analysis of newly developed degenerative spondylolisthesis in a mean twelve-year prospective study. Spine (Phila Pa 1976).

[23] Gille O, Challier V, Parent H (2014). Degenerative lumbar spondylolisthesis: cohort of 670 patients, and proposal of a new classification. Orthop Traumatol Surg Res.

[24] Chaleat-Valayer E, Mac-Thiong JM, Paquet J (2011). Sagittal spino-pelvic alignment in chronic low back pain. Eur Spine J.

[25] Zhu Z, Xu L, Zhu F (2014). Sagittal alignment of spine and pelvis in asymptomatic adults: norms in Chinese populations. Spine (Phila Pa 1976).

[26] Le Huec JC, Leijssen P, Duarte M (2011). Thoracolumbar imbalance analysis for osteotomy planification using a new method: FBI technique. Eur Spine J.

[27] Lamartina C, Berjano P (2014). Classification of sagittal imbalance based on spinal alignment and compensatory mechanisms. Eur Spine J.

[28] Xu L, Qin X, Zhang W (2015). Estimation of the ideal lumbar lordosis to be restored from spinal fusion surgery: a predictive formula for Chinese population. Spine (Phila Pa 1976).

[29] Schuller S, Charles YP, Steib JP (2011). Sagittal spinopelvic alignment and body mass index in patients with degenerative spondylolisthesis. Eur Spine J.

[30] Funao H, Tsuji T, Hosogane N (2012). Comparative study of spinopelvic sagittal alignment between patients with and without degenerative spondylolisthesis. Eur Spine J.

[31] Suzuki H, Endo K, Kobayashi H (2010). Total sagittal spinal alignment in patients with lumbar canal stenosis accompanied by intermittent claudication. Spine (Phila Pa 1976).

